# Ultrasound molecular imaging with cRGD-PLGA-PFOB nanoparticles for liver fibrosis staging in a rat model

**DOI:** 10.18632/oncotarget.21358

**Published:** 2017-09-28

**Authors:** Jiqing Xuan, Yuli Chen, Leilei Zhu, Yuan Guo, Liming Deng, Yuanyi Zheng, Zhaoxia Wang, Zhigang Wang, Meng Ao

**Affiliations:** ^1^ Department of Ultrasound, The Second Affiliated Hospital of Chongqing Medical University & Chongqing Key Laboratory of Ultrasound Molecular Imaging, Chongqing 400010, China; ^2^ Department of Ultrasound, The Affiliated Hospital of Southwest Medical University, Luzhou, 646000, China; ^3^ Institute of Ultrasound in Medicine, Shanghai Jiaotong University Affiliated Shanghai Sixth People’s Hospital, Shanghai 200233, China; ^4^ Department of Ultrasound, The Children’s Hospital of Chongqing Medical University, Chongqing 400010, China

**Keywords:** poly (lactic-co-glycolic acid), nanoparticles, ultrasound molecular imaging, hepatic fibrosis, integrin α_v_β_3_

## Abstract

Hepatic fibrosis is the only chronic liver disease process that can be reversed. Developing non-invasive and effective methods to quantitatively assess the degree of liver fibrosis is of great clinical significance and remains a major challenge. The key factors in hepatic fibrosis pathogenesis are the activation and proliferation of hepatic stellate cells that subsequently express integrin α_v_β_3_. An ultrasound (US) agent combined with a targeting peptide may be used for the early and non-invasive diagnosis of hepatic fibrosis. Herein, we report the synthesis of core-shell nanoparticles (NPs) successfully engineered by conjugation with cyclic arginine-glycine-aspartic acid (cRGD) octapeptide, allowing hepatic integrin α_v_β_3_ targeting for liver fibrosis staging. This system consists of a perfluorooctyl bromide (PFOB) liquid in the core that is stabilized with a Poly (lactic-co-glycolic acid) (PLGA) polymer shell and modified with a cRGD. These core-shell NPs (cRGD-PLGA-PFOB NPs) exhibited useful US molecular imaging features including high imaging contrast among liver fibrotic stages and the adjacent tissues. Our results indicate that the cRGD-PLGA-PFOB NPs have significant potential to distinguish different liver fibrotic stages and could be used in clinical applications.

## INTRODUCTION

Hepatic fibrosis as a pathologic healing process that results from chronic liver injury and leads to cirrhosis, liver failure, and ultimately even cancer [1, 2]. A recent study reported that early stage liver fibrosis can be reversed by efficient treatment, while advanced fibrosis and cirrhosis are usually permanent [3]. Therefore, both early detection and continuous monitoring of liver fibrosis have important clinical implications for patients with chronic liver diseases.

Hepatic fibrosis is characterized by the activation and proliferation of hepatic stellate cells (HSCs) [4, 5] following liver injury. When the liver is injured due to viral infection or hepatic toxins, quiescent HSCs (qHSCs) receive signals secreted from damaged hepatocytes and immune cells, causing them to transdifferentiate into activated hepatic stellate cells (aHSC), and this is accompanied by increased production and deposition of extracellular matrix (ECM) proteins [6]. The most extensively studied is integrin α_v_β_3_, which binds to ECM protein by recognizing the three amino acid sequence of arginine-glycine-aspartic acid (RGD) [7-9]. Additionally, hepatic integrin α_v_β_3_ expression is reportedly increased with liver fibrosis development and progression [5, 10, 11]. Therefore, hepatic integrin α_v_β_3_ is a useful target for monitoring the fibrogenic process.

Liver biopsy is considered the gold standard to stage liver fibrosis [12]; however, it is an invasive procedure with significant complications and not usually accepted by patients. Moreover, this invasive examination can produce a false-negative result, making it difficult to observe progression dynamics and compare follow-up findings. In this regard, it is crucial to develop a non-invasive, simple, and accurate method to dynamically monitor and evaluate the degree of liver fibrosis. Conventional imaging techniques such as ultrasound (US), computed tomography, and magnetic resonance are often used to diagnose liver cirrhosis and related complications, but these modalities have low sensitivities to diagnose early liver fibrosis. Molecularly targeted imaging techniques are therefore needed to improve diagnostic sensitivity [13, 14].

US is a widely used clinical diagnostic modality and has advantages of being cost effective, non-invasive, non-ionizing, and allowing real-time temporal resolution with various imaging modes [15, 16]. Targeted US contrast agents have been prepared by attaching targeting ligands to the lipid, protein, or poly shell coating of gas-filled microbubbles. After intravenous administration, these molecular probes aggregate in the target tissues to enhance imaging [17]. However, currently used microscale US contrast agents (microbubbles) have diameters of 1-10 μm and cannot pass through the vasculature to certain targets, and their limited stability hampers their use as molecularly targeted agents [18, 19]. Nanotechnology can play a pivotal role in improving targeted molecular imaging agents. Nanoparticles (NPs) of liquid perfluorocarbons (PFCs) in water have interesting US properties similar to gaseous PFCs, particularly at high frequencies [20]. As long as NPs are highly concentrated, they have good US enhancement of the blood pool *in vivo* and can be potentially adapted for targeted imaging. Since angiogenesis and neovascularization are significantly increased during liver fibrosis development [2, 21, 22], NP contrast agents can pass through the vascular endothelia gap (nanometric) of new vessels and be retained in the ECM for a long time, offering the potential for strong and selective accumulation of the molecular imaging agent.

Poly lactic-co-glycolic acid (PLGA) is a preferential candidate as a carrier material for targeting microbubbles because of its high stability, biocompatibility, and biodegradability, which ensure good acoustic properties and prolonged circulation time. Moreover, acoustic characteristics can be controlled by adjusting the chemical composition and molecular weight of the polymer. PLGA has been approved by the United States Food and Drug Administration and has been used as a suture material in humans for many years. Due to their enhanced drug loading capacity, biological stability, and sustainable circulation time *in vivo*, organic PLGA NPs have recently been employed in applications of drug delivery, tissue engineering, and molecular imaging [23-25]. A biocompatible and biodegradable polymer shell would improve NP stability compared with microbubbles stabilized by a monomolecular layer, while a perfluorocarbon core could provide echogenicity [26].

Based on the above considerations, we sought to modify the surface chemistry of PLGA NPs encapsulating perfluorooctyl bromide (PFOB) using the cyclic arginine-glycine-aspartic acid (cRGD) peptide. These modified NPs (cRGD-PLGA-PFOB NPs) could be used as an US contrast agent to specifically target integrin α_v_β_3_ expressed on activated HSCs at different stages of fibrosis. In this study, PLGA-PFOB NPs were successfully prepared by a double emulsion method followed by solvent evaporation [27, 28]. We further modified the PLGA-PFOB NPs with cRGD so the targeted cRGD-PLGA-PFOB NPs can actively recognize and be efficiently taken up by activated HSCs. We generated a rat liver fibrosis model induced by carbon tetrachloride (CCl_4_) and detected integrin α_v_β_3_ expression at different stages of liver fibrosis. Finally, we performed *in vivo* experiments to explore the feasibility of targeted US molecular imaging as a non-invasive approach to stage liver fibrosis.

## RESULTS

### Characterization of PLGA-PFOB NPs and cRGD-PLGA-PFOB NPs

**Figure 1:**
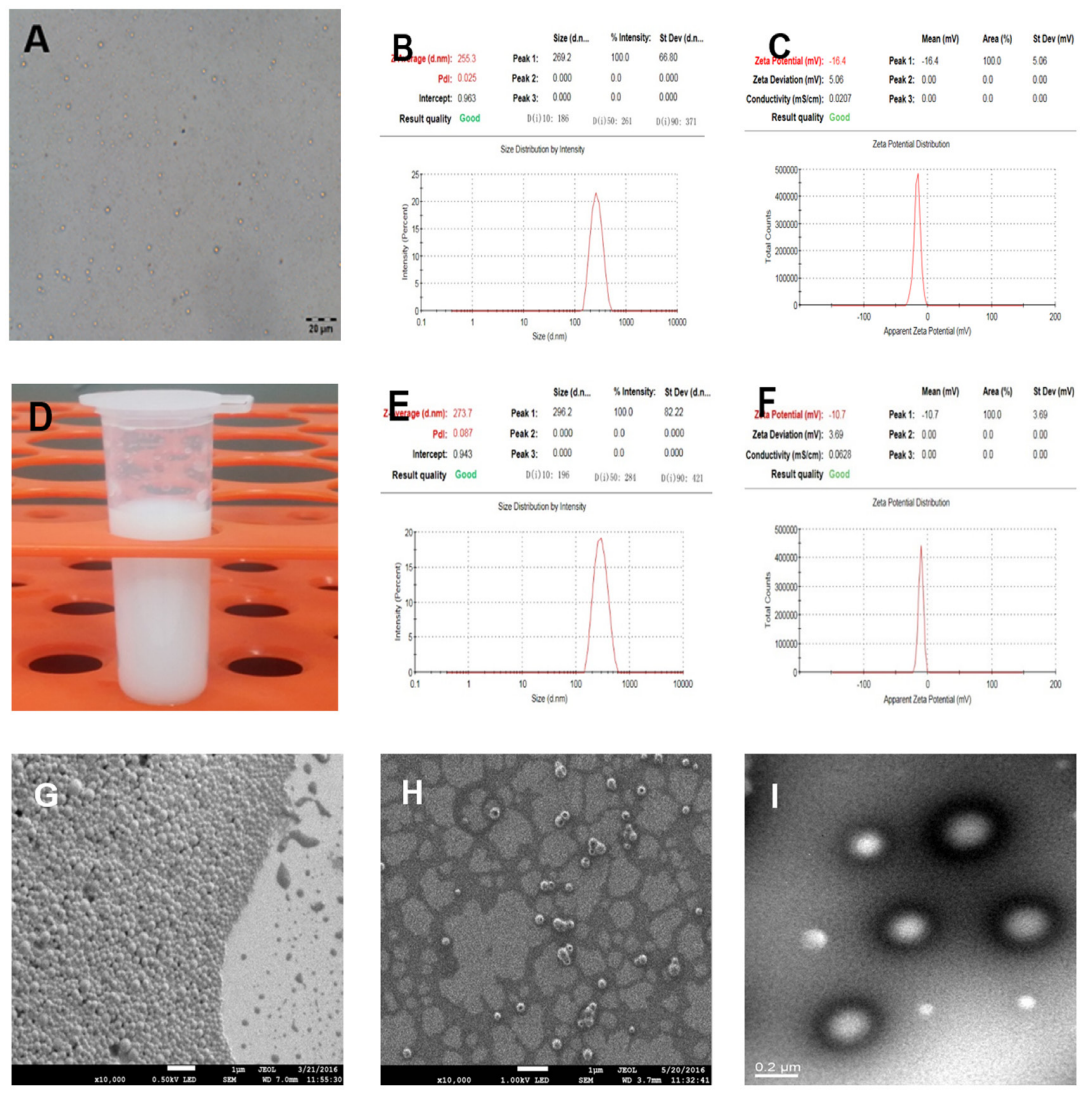
Characterization of cRGD-PLGA-PFOB NPs and PLGA-PFOB NPs **(A)** Optical microscope images of PLGA-PFOB NPs. **(B)** and **(C)** The size and zeta distribution of PLGA-PFOB NPs measured by dynamic light scattering (DLS) and electrophoretic light scattering (ELS). **(D)** Digital photos of PLGA-PFOB NPs at a concentration of 50 mg/mL in DI water. **(E)** and **(F)** The size and zeta distribution of cRGD-PLGA-PFOB NPs measured by DLS and ELS. **(G)** and **(H)** SEM image of PLGA-PFOB NPs at different concentrations. Scale, 1 μm. **(I)** TEM image of typical NPs; because of the difference in electronic density, the PFOB liquid core appears gray, while the polymeric shell seems darker. Scale, 0.2 μm.

PLGA-PFOB NPs suspensions were successfully prepared with high stability and did not exhibit morphologic changes when kept in deionized water at room temperature for more than 3 days. PLGA-PFOB NPs suspension were milky white in color (Figure 1D). Under light microscopy, PLGA-PFOB NPs were small spheres distributed evenly in the suspension (Figure 1A). The mean particle size and zeta potential were 255.3 nm and -16.4 mV, respectively (Figure 1B and 1C), while those of cRGD-PLGA-PFOB NPs were 273.7 nm and -10.7 mV, respectively (Figure 1E and 1F). Scanning electron microscopy (SEM) images showed that most NPs are spherical with smooth surfaces (Figure 1G and 1H). To investigate whether PFOB could be efficiently encapsulated into NPs, diluted NP suspension samples were observed by transmission electron microscopy (TEM). They appeared as spherical particles with a core-shell structure because there is an obvious electronic density difference between the core and shell (Figure 1I).

### Conjugation efficiency of cRGD on PLGA-PFOB NPs

**Figure 2:**
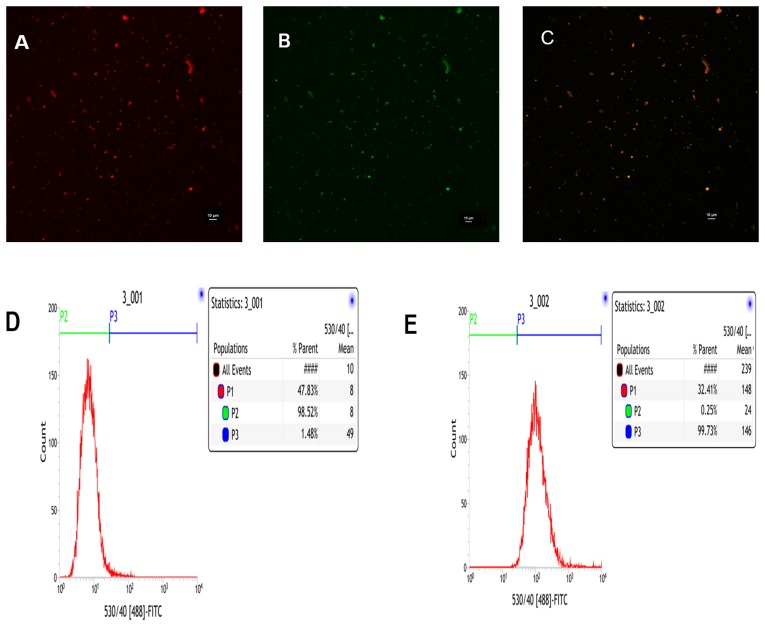
CLSM and FCM images of the PLGA-PFOB NPs modified with cRGD cRGD-PLGA-PFOB NPs modified with cRGD were prepared in the presence of Nile Red to stain for PLGA-PFOB NPs, and a FITC label was used for cRGD peptides. **(A)** Nile Red. **(B)** FITC. **(C)** Merge. **(D)** FCM image of PLGA-PFOB NPs. **(E)** FCM image of PLGA-PFOB NPs modified with FITC-labeled cRGD peptide.

To visualize binding of cRGD to PLGA-PFOB NPs and to provide the PLGA-PFOB NPs with fluorescence imaging capability, we used Nile Red-dyed PLGA-PFOB NPs and FITC-labeled cRGD. The cRGD peptides were uniformly conjugated onto PLGA-PFOB NPs as demonstrated with confocal laser scanning microscope (CLSM) imaging (Figure 2). Nile Red-dyed PLGA-PFOB NPs emitted red fluorescence (Figure 2A), FITC-labeled cRGD emitted green fluorescence (Figure 2B), and overlay fluorescence images were homogenously bright yellow (Figure 2C), indicating that cRGD were uniformly conjugated onto PLGA-PFOB NPs. We chose Nile Red as a fluorescent marker to the organic solution prior to emulsification because it colors the hydrophobic polymer but not PFCs. CLSM images show PLGA-PFOB NPs had a uniform size and were monodispersed.

The flow cytometry (FCM) results show that ∼99.73% of the targeted cRGD-PLGA-PFOB NPs were FITC positive (Figure 2E), compared to only 1.48% of the PLGA-PFOB NPs (Figure 2D), which demonstrates good conjugation between cRGD peptides and PLGA-PFOB NPs.

### *In vitro* US imaging

*In vitro* US enhancement of cRGD-PLGA-PFOB NPs was evaluated at concentrations of 50, 25, and 12.5 mg/mL. The cRGD-PLGA-PFOB NP suspensions appear brighter than background in non-linear (THI) mode, and the signal enhances in a concentration-dependent fashion (Figure 7A).

### Cytotoxicities of PLGA-PFOB NPs and cRGD-PLGA-PFOB NPs

**Figure 3:**
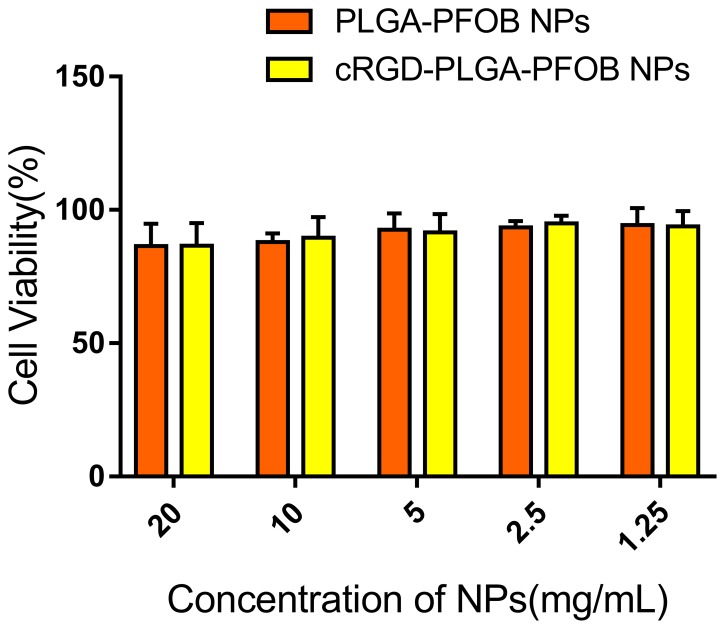
*In vitro* cytotoxicity of PLGA-PFOB NPs and cRGD-PLGA-PFOB NPs in BRL-3A hepatic cells Cells were treated with different concentrations of NPs for 24 h.

As shown in Figure 3, BRL-3A cell viabilities were 86.6 ± 8.5%, 89.5 ± 7.8%, 91.5 ± 7.0%, 94.9 ± 2.9%, and 93.8 ± 5.8% for groups cultured with cRGD-PLGA-PFOB NPs at concentrations of 20, 10, 5, 2.5, and 1.25 mg/mL, respectively. NPs had no significant influence on cell viability considering the same polymer concentration. There was no definite correlation between PLGA-PFOB NP or cRGD-PLGA-PFOB NP concentration and cell viability, and we did not note significant differences among PLGA-PFOB NP or cRGD-PLGA-PFOB NP groups compared with control (*p* > 0.05), indicating low cytotoxicity and favorable biocompatibility of cRGD-modified PLGA-PFOB NPs.

### Acute biosafety of cRGD-PLGA-PFOB NPs

We injected cRGD-PLGA-PFOB NPs suspensions into rats to investigate their acute biosafety *in vivo*. After injecting a dosage of 0.5 mL/100 g from 50 mg/mL suspensions, there were no obvious abnormalities in any of the rats. The serum biochemical indicator levels (Table 1) confirmed that liver and kidney function were not significantly altered (*p* > 0.05) at any of the post-injection time points (1, 3, 7, and 14 days). These results demonstrate that intravenously injected cRGD-PLGA-PFOB NPs have good biocompatibility.

**Table 1:** Serum biochemical indicators after cRGD-PLGA-PFOB NP injection (50 mg/mL)

	Pre-injection	1d	3d	7d	14d
TP (g/L)	72.70±9.73	75.67±10.62	79.32±11.21	75.82±7.47	72.49±8.03
ALB (g/L)	35.83±5.38	37.25±5.81	34.79±4.72	38.78±8.11	36.06±11.15
ALT (U/L)	48.50±9.31	52.17±9.35	56.50±6.28	50.16±10.46	50.50±12.88
AST (U/L)	108.67±18.39	113.33±15.22	107.33±17.85	111.00±20.65	113.17±17.03
DBIL (μmol/L)	0.50±0.32	0.65±0.33	0.67±0.30	0.77±0.40	0.88±0.40
TBIL (μmol/L)	3.47±1.61	4.30±1.76	4.30±2.33	3.50±0.70	4.43±2.95
BUN (mmol/L)	8.00±1.63	7.79±1.78	7.69±1.27	8.67±1.62	7.33±0.77
SCr (μmol/L)	29.67±3.87	32.67±9.54	33.30±7.22	31.47±5.32	29.30±9.14

Note: The same indicators at different time points for pairwise comparisons (all *p*> 0.05).

### Cell-targeting ability of cRGD-PLGA-PFOB NPs *in vitro*

**Figure 4:**
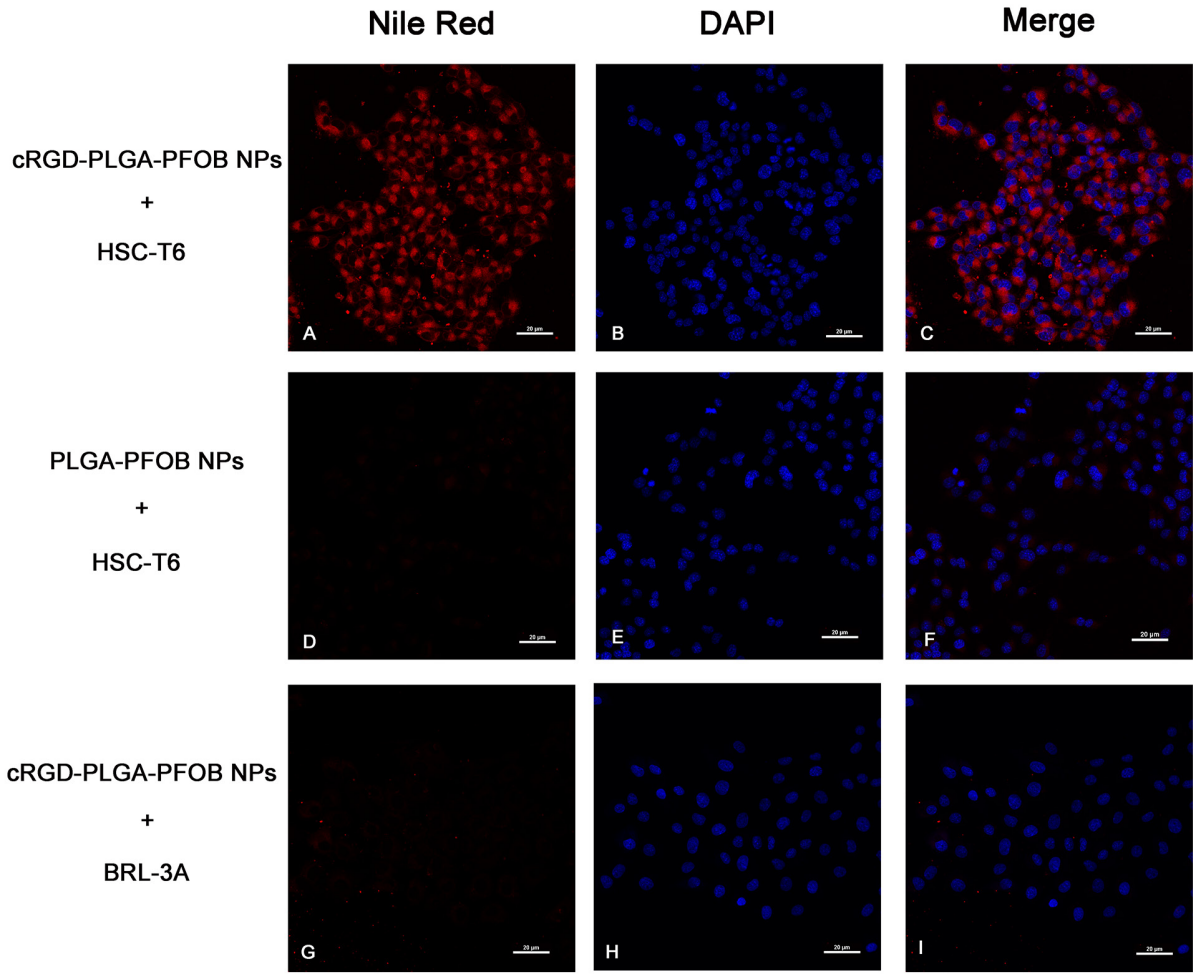
*In vitro* targeting performance of cRGD-PLGA-PFOB NPs to HSC-T6 cells (magnification ×400) CLSM images of HSC-T6 cells after 30-min treatment with cRGD-PLGA-PFOB NPs **(A-C)** or PLGA-PFOB NPs **(D-F)**. To determine the intracellular location of Nile Red-doped NPs (red fluorescence), cell nuclei were counterstained with DAPI (blue fluorescence). NPs were mainly present in the cytomembranes and cytoplasm of HSCs. The absence of signal in the nucleus indicated they could not pass through the nuclear membrane. BRL-3A cells treatment with cRGD-PLGA-PFOB NPs **(G-I)**. Scale, 20 μm.

By using a CLSM, stronger and more abundant red fluorescence could be observed in the cytoplasm of HSC-T6 cells at 30 min after treatment with cRGD-PLGA-PFOB NPs (Figure 4A-4C) compared to PLGA-PFOB NPs (Figure 4D-4F). Less red fluorescence was observed in the cytoplasm of BRL-3A cells 30 min after treatment with cRGD-PLGA-PFOB NPs (Figure 4G-4I), indicating greater cell targeting and affinity efficiency of cRGD-PLGA-PFOB NPs to HSCs.

### Liver fibrosis stage analysis

**Figure 5:**
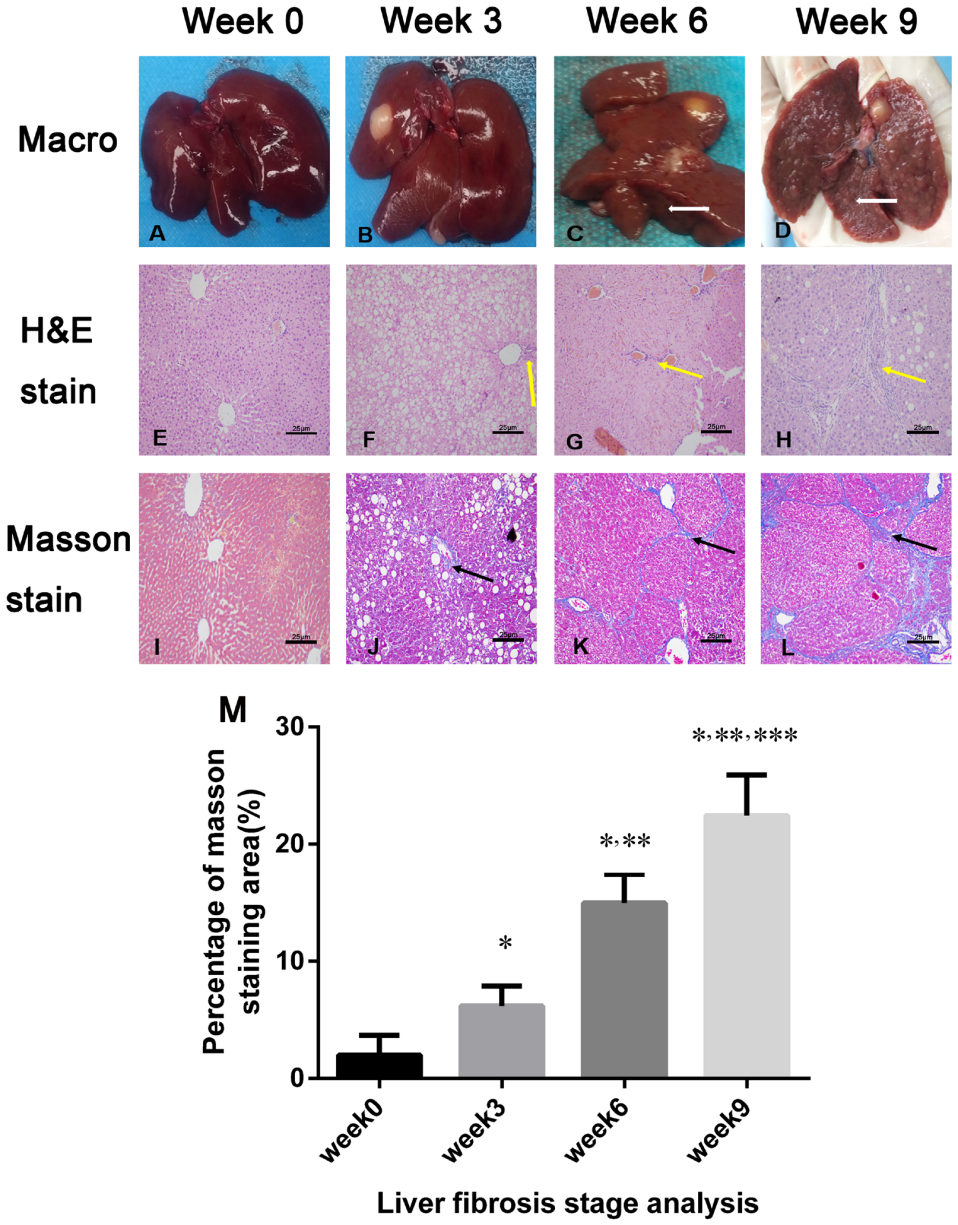
Macroscopic inspections and pathological examination of the fibrosis liver after 0, 3, 6, and 9 weeks of CCl4 induction **(A-D)** Macroscopic inspection showed fibrous septa and nodules in the liver after 6 weeks of CCl_4_ induction (white arrows in C and D). **(E-H)** Representative micrographs of hepatic histology stained with H&E (×200), and increased collagen fibers with liver fibrosis progression (yellow arrow in F, G, and H). Scale, 25 μm. **(I-L)** Liver specimens (×200) were stained with Masson’s trichrome to reveal collagen fibers after 0, 3, 6, and 9 weeks of CCl_4_ induction; blue-stained areas represented collagen fibers (black arrows in J, K, L). Scale, 25 μm. **(M)** Masson’s trichrome staining (fibrosis) was compared among the liver fibrosis and control groups (n = 3 each). For semiquantitative analysis of liver fibrosis, 10 fields (×200) from each section were randomly selected, recorded, and measured. ^*^p < 0.05 versus week 0 group, ^**^p < 0.05 versus week 3 group, ^***^p< 0.05 versus week 6 group.

After 3 weeks of CCl_4_, administration there was no significant macroscopic changes in the liver. With longer CCl_4_ treatment, the liver texture hardened, and fibrous tissues and nodules were observed on the surface (Figures 5C-5D). Hepatocyte injury such as ballooning degeneration were observed in the 3-week group (Figure 5F and 5J), and the percentage of fibrotic area was 6.40 ± 2.16%. During longer administration, collagen deposition was increased with liver fibrosis progression. The percentage of fibrotic area significantly increased to 14.62 ± 2.90% in the 6-week group and 22.84 ± 4.14 % in the 9-week group, respectively (both *p* < 0.05) (Figure 5M).

### Integrin α_v_β_3_ expression in fibrotic liver

**Figure 6:**
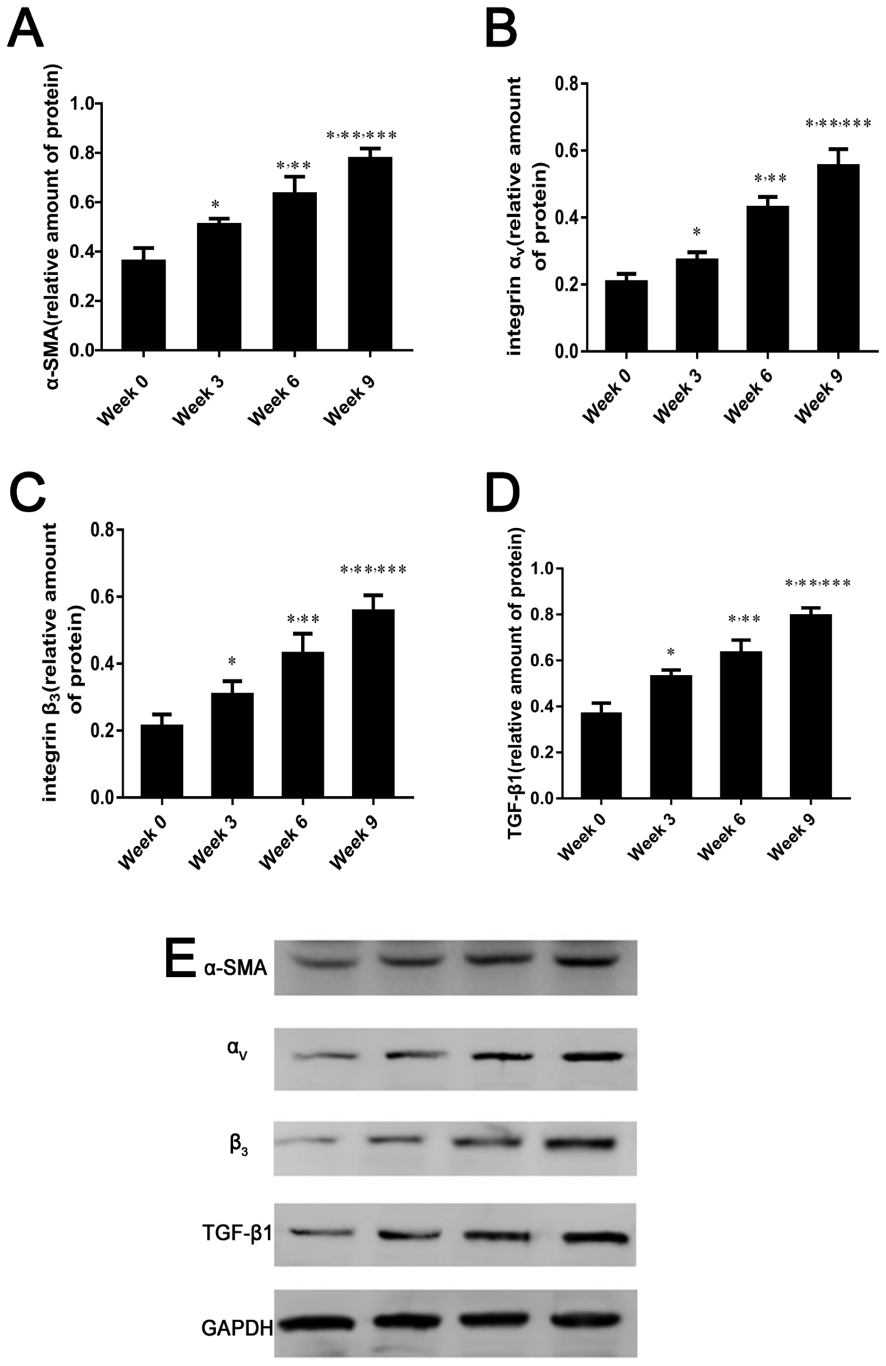
α-SMA, integrin αvβ3 and TGF-β1 expression in fibrotic liver **(A-E)** Relative protein amounts of α-SMA, α_v_ and β_3_ integrin subunits, and TGF-β_1_ by western blot in rat liver induced by CCl_4_ for 0, 3, 6, or 9 weeks. Protein levels are represent as the ratio to GAPDH. Data are expressed as means ± standard deviation (SD) (n=4 per group). In all panels ^*^p < 0.05 versus week 0 group, ^**^p < 0.05 versus week 3 group, ^***^p < 0.05 versus week 6 group.

With increased liver fibrosis, the protein levels of α-smooth muscle actin (SMA), α_v_ and β_3_ integrin subunits, and transforming growth factor-β1 (TGF-β_1_) were remarkably increased and were highest in the 9-week group (*p* < 0.05 for comparisons among all groups, Figure 6).

### *In vivo* US imaging and image analysis

**Figure 7:**
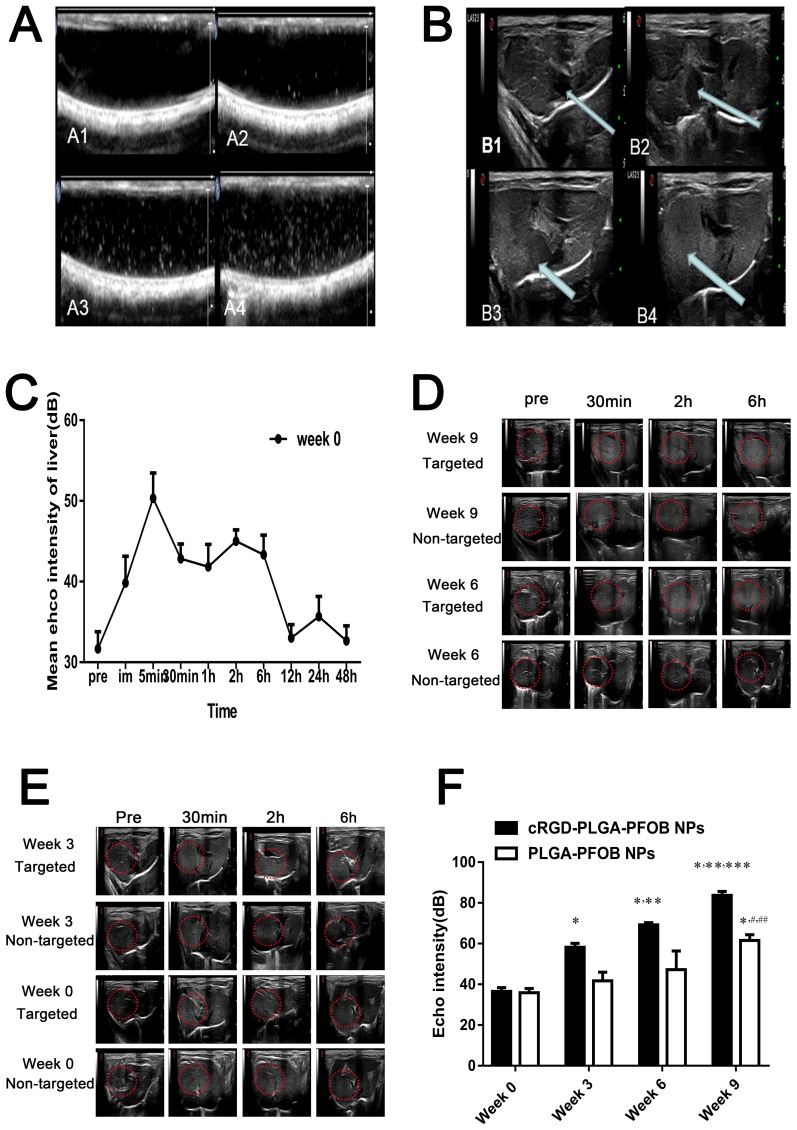
*In vitro* and *in vivo* US imaging **(A)** US images obtained *in vitro* in non-linear mode (THI). (**(A1)** Water in a silicone tube appears dark; **(A2-4)** concentrations of 12.5, 25, and 50 mg/mL in DI water). **(B)** US imaging of normal rat liver before and after PLGA-PFOB NP injection (**(B1)** Liver before injection of PLGA-PFOB NPs, the inferior vena cava (arrow) appears dark. **(B2)** The inferior vena cava (arrow) presents immediate enhancement after PLGA-PFOB NP injection. **(B3, B4)** The inferior vena cava and liver parenchyma present subsequent enhancement). **(C)** Echo intensity of the liver parenchyma in the week 0 group within 48 hours after PLGA-PFOB NP injection. **(D)** and **(E)** Contrast-enhanced liver US images of different groups after injection of targeted contrast agents (cRGD-PLGA-PFOB NPs) or non-targeted contrast agents (PLGA-PFOB NPs). **(F)** EI values were compared among the liver fibrosis and control groups after injection of cRGD-PLGA-PFOB NPs or PLGA-PFOB NPs. ^*^p < 0.05 versus week 0 group, ^**^p < 0.05 versus week 3 group, ^***^p < 0.05 versus week 6 group, ^#^*p* < 0.05 versus week 0 or 3 group, ^##^ *p* < 0.05 versus week 6 group.

To verify the contrast enhancement ability of PLGA-PFOB NPs suspensions *in vivo*, high-frequency US was performed in control rats. Figures 7B shows the typical enhancement process of the rat liver before and after PLGA-PFOB NP injection. Initially, the large vessels within the liver and inferior vena cava appeared dark (Figure 7B1, arrow). After intravenous injection of PLGA-PFOB NPs (1.1 ml of a 50 mg/mL suspension), the inferior vena cava presented immediate enhancement (Figure 7B2), and large vessels within the liver and liver parenchyma were subsequently enhanced (Figure 7B3 and 7B4). To investigate contrast enhancement duration and peak value of PLGA-PFOB NPs contrast agents, the echo intensity (EI) in the liver was detected before and at 0 min, 5 min, 30 min, 1 h, 2 h, 6 h, 12 h, 24 h, and 48 h after PLGA-PFOB NP injection. As shown in Figure 7C, the EI (n=6) in the liver peaked at 5 min, but contrast enhancement persisted for ∼6 h. After that, the liver EI decreased significantly and reduced to the baseline value at 12 h. In subsequent experiments, we only acquired and analyzed images before injection and within 6 h after injection.

To further validate the active targeting efficiency of cRGD-PLGA-PFOB NPs suspensions, high-frequency US was performed in rats with induced fibrosis. As shown in Figures 7D, 7E and 7F, rats in the fibrosis group (3 weeks, 6 weeks, and 9 weeks after CCl_4_ injection of the cRGD-PLGA-PFOB NPs) had significantly higher EI in the liver at 6 h than those injected with PLGA-PFOB NPs (*p* < 0.001). After cRGD-PLGA-PFOB NP injection, the EI for rats with advanced liver fibrosis (9-week group) was significantly higher than that of rats with mild liver fibrosis (3- and 6-week groups) or normal liver (0-week group). After PLGA-PFOB NP injection, the EI of the 9-week group was significantly higher than the 0-, 3-, and 6-week groups (*p*< 0.05). However, no changes in EI were found among the 0-, 3-, and 6-week groups after PLGA-PFOB NP injection.

## DISCUSSION

Targeted cRGD-PLGA-PFOB NPs US contrast agents were successfully prepared using a double emulsion method, achieving uniform size and good dispersion as illustrated in Figures 1A and 1B. In previous studies, several PFCs were evaluated for encapsulation with polymeric shells for developing targeted US contrast agents with a small NP size [29]. We chose PFOB as the liquid perfluorocarbon core of the NP since no toxicity has been reported for this chemical and it has a high boiling point [30, 31]. The method used to obtain NPs composed of a solid PLGA shell encapsulating a liquid PFOB core was derived from the technique described by Loxley and Pisani [32, 33].

**Figure 8:**
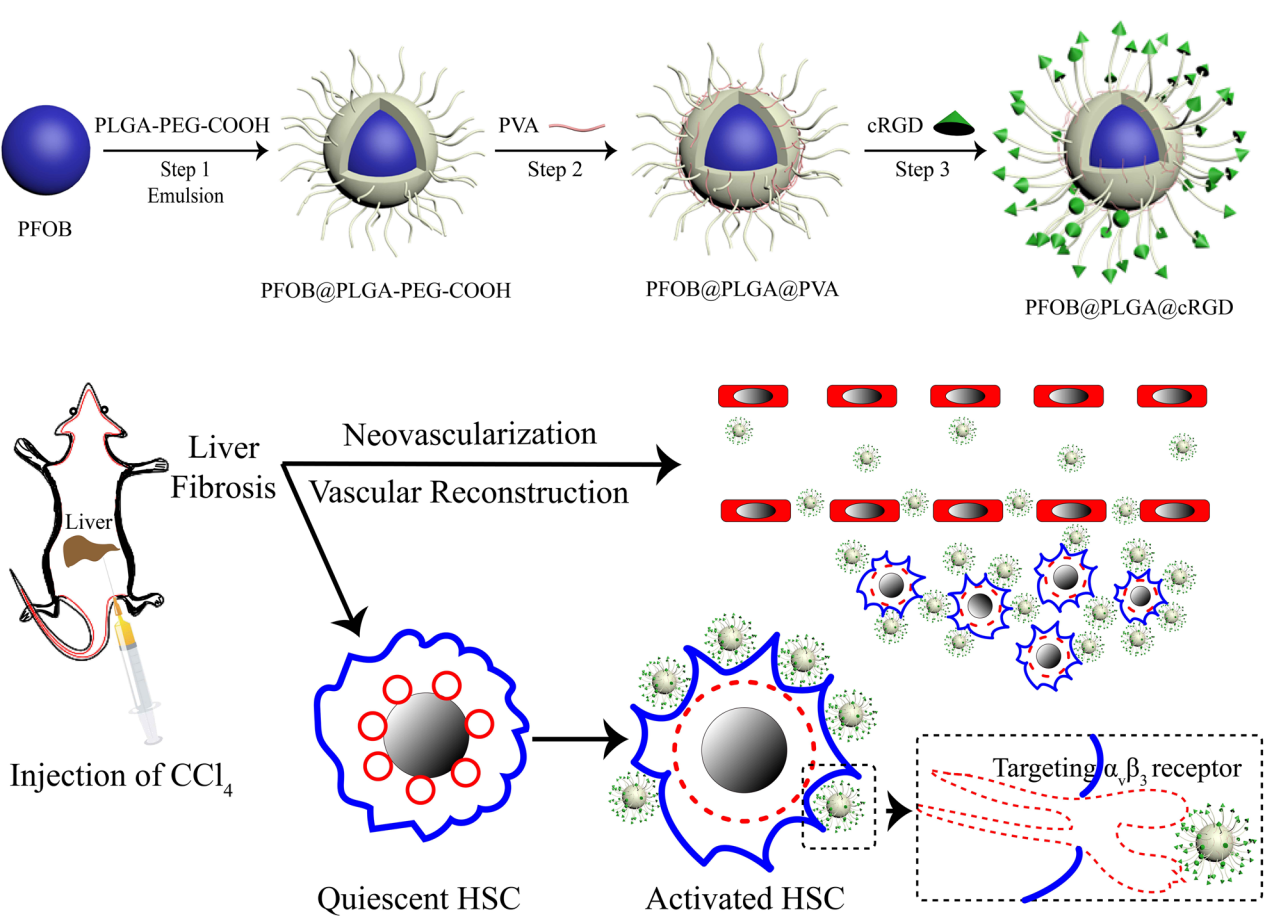
Schematic illustration of cRGD-PLGA-PFOB NPs for targeted molecular imaging The diagram depicts the fabrication process and targeting capabilities to integrin α_v_β_3_ expressed by activated HSCs.

To achieve a targeted NP system, we applied carbodiimide covalent coupling to modify the PLGA-PFOB NP surface with cRGD peptides. This covalent binding mode is frequently used for coupling targeted delivery systems with bioactive molecules such as DSPE-PEG-COOH immunoliposomes [34] and PLGA-PEG-COOH polymer targeting micro/nanoparticles [35, 36]. The schematic is shown in Figure 8. PLGA-PEG-COOH in NP film-forming materials is an amphipathic substance, and its hydrophilic carboxy moieties were connected with the NP surface through PEG chains. Therefore, hydrophilic carboxy was activated by the EDC/NHS coupling activator, and cRGD was more likely to be conjugated due to the presence of a PEG chain that reduced steric hindrance and facilitated the coupling reaction. The cRGD peptide consists of eight amino acids connected with disulfide bonds. Therefore, the amino group of the cRGD peptide and activated carboxyls of PLGA-PFOB NPs were firmly bonded with covalent bonds. This binding was stable and effective, and was not significantly affected by shear flow in blood circulation. As shown with CLSM and FCM experiments *in vitro*, cRGD peptides were successfully conjugated onto PLGA-PFOB NPs. Rat hepatocytes were viable after treatment with the cRGD-PLGA-PFOB NPs/PLGA-PFOB NPs at various concentrations, even up to 20 mg/mL, indicating low cytotoxicity and favorable biocompatibility of cRGD-modified PLGA-PFOB NPs as a US contrast agent for *in vivo* applications.

RGD-containing cyclic peptides have been proposed as integrin α_v_β_3_ antagonists to recognize integrin α_v_β_3_ on activated HSCs [11, 37, 38]. We investigated the targeting efficiency of cRGD-PLGA-PFOB NPs to HSCs with *in vitro* cell experiments. Direct CLSM observation revealed that after incubating with cRGD-PLGA-PFOB NPs/PLGA-PFOB NPs for 30 min, HSC-T6 took up more targeted cRGD-PLGA-PFOB NPs than PLGA-PFOB NPs. This indicated that the targeted cRGD-PLGA-PFOB NPs specifically bound to integrin α_v_β_3_ on activated HSCs, providing a theoretical basis for targeted imaging *in vivo*.

Several researchers have demonstrated that α-SMA is a characterized marker of activated HSCs, which are the cardinal cells expressing integrin α_v_β_3_ in the liver sinusoid areas affected by fibrosis [5, 39]. In addition, TGF-β1 is considered a key factor in accelerating hepatic fibrosis because it is released from activated HSCs and further stimulates them [40]. In this study, we found that integrin α_v_β_3_, α-SMA, and TGF-β1 expression increased with the progression of liver fibrosis, which is similar to previously reported results [41, 42]. Moreover, most integrin α_v_β_3_ is expressed in activated HSCs; much less α_v_β_3_ is expressed in qHSCs, hepatocytes, and other non-parenchymal cells [11]. These findings provide evidence that integrin α_v_β_3_ expression correlated well with the degree of liver fibrosis. Therefore, hepatic integrin α_v_β_3_ represents a target for monitoring fibrogenic processes.

The *in vivo* imaging experiments demonstrated that the cRGD-PLGA-PFOB NPs/PLGA-PFOB NPs contrast agent can enhance US imaging. Before contrast agent injection, there were no significant differences in EI in B-mode among the different CCl_4_ groups compared with the normal control group, which only showed morphology changes with a rough echo. After contrast agent injection, increased US EIs were successively observed in the inferior vena cava and large vessels within the liver and liver parenchyma. Through dynamic observation, we found the EI of liver parenchyma peaked at 5 min and maintained at a relatively high level within 6 h. At 6 h after agent injection, the liver EI gradually decreased and returned to the level of pre-injection after 12 h (Figure 7C). We consider that the peak EI at 5 min is a flow effect of cRGD-PLGA-PFOB NPs/PLGA-PFOB NPs in the liver perfusion peak and did not allow enough targeting effect time. Therefore, 6 h after injection was selected as the observation time for subsequent *in vivo* targeted transportation experiments of cRGD-PLGA-PFOB NPs. From the liver images before PLGA-PFOB NP injection (first column, Figure 7D, 7E), there was no difference in EI among the different degrees of fibrosis by visual inspection or software analysis. Interestingly, after intravenous cRGD-PLGA-PFOB NP injection, the echo of rats livers were enhanced to levels significantly stronger than those before injection or those in animals injected with PLGA-PFOB NPs. As shown in Figure 7F, quantitative analysis revealed a dose-dependent increase of EI of livers in CCl_4_-treated groups from 58.17 ± 1.94 to 69.21 ± 1.11 to 83.67 ± 2.17, which was significantly higher than that of the control group (36.5 ± 1.87). Notably, EI increased with the degree of liver fibrosis. The significant differences in EI indicated that the cRGD-PLGA-PFOB NPs were specifically recognized and taken up by activated HSCs. This suggests that cRGD-PLGA-PFOB NPs can provide useful information for assessing the degree of liver fibrosis.

To avoid non-specific uptake of NPs by Kupffer cells affecting the EI results, we included a control group of rats with a similar fibrosis stage that were injected with the same dose of PLGA-PFOB NPs. There were only significant differences in EI for the week 9 group compared with week 0, 3, and 6 groups. This phenomenon could be explained by three reasons. Firstly, macrophages in the liver increased with the development of liver fibrosis, and these may phagocytose more NPs. Secondly, angiogenesis and revascularization increased along with fibrosis severity [23] [43]. These newly formed vessels have poorly aligned defective endothelial cells with wide fenestrations, lack a smooth muscle layer, and a wider lumen. The abnormal architecture usually lacks effective lymphatic drainage. For these reasons, NPs tend to accumulate in advanced fibrotic liver tissues much more than in mildly fibrotic and normal liver tissues.

Contrast-enhanced US with microbubbles has been reported as a useful method to evaluate liver fibrosis severity or diagnose hepatic cirrhosis [44-46]. However, most researchers assessed the degree of hepatic fibrosis by observing hemodynamic changes after contrast agent injection, such as the transit times of the hepatic artery and portal vein, to assess overall fibrosis. Microbubbles are a blood pool contrast agent, which can only enhance intravascular imaging. After injection, the time of liver enhancement imaging is short, usually only a few minutes. The comparatively long half-life and greater time of liver enhancement imaging are advantages of our prepared PLGA-PFOB NPs. They also have stronger penetrability and can access the tissue space to enhance target tissue imaging. By analyzing the EI of the local area of interest in the liver, we can evaluate the degrees of whole-liver and regional fibrosis. Collectively, our *in vitro* and *in vivo* results indicate that cRGD-PLGA-PFOB NPs are effective targeting probes for US molecular imaging and can specifically bind to integrin α_v_β_3_ in activated HSCs. It could be a promising tool to non-invasively distinguish different stages of liver fibrosis.

## MATERIALS AND METHODS

### NP preparation and characterization

PLGA NPs were prepared by a double emulsion solvent (water/oil/water) evaporation process to obtain NPs with a modified polymeric shell encapsulating perflurooctyl bromide (PFOB). Briefly, 100 mg PLGA-PEG-COOH (LA/GA = 50:50, MW = 12,000, Daigang, China) was dissolved into 4 mL methylene chloride along with 60 μL PFOB. The mixture was emulsified using an ultrasonic probe (SONICS & MATERALS Inc., Newtown, CT, USA) at 130 W for 4 min (5 s on and 5 s off) in a 50-mL centrifuge tube placed over ice. After adding 10 mL poly (vinyl alcohol) (PVA; MW = 25,000; Sigma, St. Louis, MO, USA) solution (4% w/v), the solution was emulsified within 3 min for a second time. Then, 20 ml isopropanol solution (2% w/v) was added. Methylene chloride and isopropanol were then evaporated by magnetic stirring for about 6 h at 300 rpm at room temperature. After full evaporation of the solvents, NPs were collected by centrifugation (10,000 g, 7 min) (Eppendorf AG, 5804R, Hamburg, Germany) and washed with deionized (DI) water. For confocal microscopy, Nile Red was added to the organic solution prior to emulsification. Typically, ∼100 μL of a concentrated Nile Red solution (0.057 mg/mL in methylene chloride) was added to the organic solution [28]. The supernatant containing the surfactant was discarded, and the NPs were re-suspended with DI water (2 mL) by vortexing (30s). Finally, the suspension was collected and stored at 4°C for further use.

The average size and zeta potential of NPs were measured using the Laser Particle Size Analyzer System (Zeta SIZER 3000HS: Malvern, UK). Diameter measurement was repeated three times. Results are expressed as mean ± SD. The morphological and structural characteristics of the NPs were observed with optical microscopy (CKX41, Olympus, Tokyo, Japan), SEM (JEOL JSM-7800F, Tokyo, Japan) and TEM (H-7500, Hitachi, Tokyo, Japan).

### Synthesis of cyclic RGD peptide and conjugation to PLGA-PFOB NPs

The sequence of cyclic RGD peptide (Cys-Gly-Arg-Gly-Asp-Ser-Pro-Cys or C^*^GRGDSPC^*^) was selected based on its cell adhesion mediated by activated HSCs [37]. Chinapeptides Co., Ltd (Shanghai, China) synthesized the cyclic RGD peptide, and a subset were labeled with fluorescein isothiocyanate (FITC) for fluorescence detection. Cyclic RGD peptide immobilization on PLGA-PFOB NPs was completed through the amide condensation reaction. 1-ethy-3-(3-thylaminopropyl) carbodiimide hydrochloride (EDC) (16 mg, 0.083 mmol), N-Hydroxysuccinimide (NHS) (48 mg, 0.417 mmol) and 100 mg of the above-obtained PLGA-PFOB NPs were dissolved in MES (2-ethanesulfonic acid) buffer (pH = 5.5). The mixed solution was placed in a 10-mL centrifuge tube that was oscillated (120 rpm) and incubated for 40 min at 20°C. Then, the mixture was centrifuged at 10,000 g and washed three times to remove the extra surfactants and other salts. Subsequently, the above reactants were re-dispersed in MES buffer (pH = 8.0) containing cRGD peptide (6.6 mg, 0.0083 mmol) and then oscillated (120 rpm) overnight at the same temperature. After the reaction, free peptides and extra salts were discarded by centrifugation. Finally, the purified cRGD peptide-modified PLGA-PFOB NPs (targeted PLGA-PFOB NPs) were stored at 4°C for further experiments.

### Determination of the conjugation efficiency of cycle RGD peptide to PLGA-PFOB NPs

To verify that cRGD conjugated onto the PLGA-PFOB NP surface, a diluted NP suspension was placed on the bottom of a glass Petri dish (NEST, Wuxi, China) and examined with a CLSM (A1R, Nikon, Tokyo, Japan). Nile Red (red) was added as a fluorescent marker to stain the hydrophobic polymer. The PLGA-PFOB NPs dyed with Nile red were excited at 543 nm and observed at 560 nm. The cRGD labeled with FITC (green) were excited at 488 nm and observed at 525 nm. The dispersion liquid of PLGA-PFOB NPs modified with FITC-labeled cRGD peptide was detected using flow cytometry (FACScalibur, Becton Dickinson, Franklin Lakes, NJ, USA) with the excitation setting at 488 nm, and blank PLGA-PFOB NPs were used as a control. The data were analyzed with CellQuest software (Becton Dickinson).

### Cell and animal models

The rat hepatic stellate cell line (HSC-T6) and rat hepatocyte line (BRL-3A) were purchased from Cell Bank (PROCELL LIFE SCI & TECH CO., LTD, Wuhan, China). All animal experimental protocols were approved by the Ethical Committee of Chongqing Medical University. Eight-week-old inbred female Sprague Dawley rats (6-8 weeks old, body weight 200 ± 20 g) were obtained from the Laboratory Animal Center of Chongqing Medical University and fed standard laboratory rat chow on a 12-hour light/dark cycle with free access to water and food. In order to induce liver fibrosis, rats were subcutaneously injected with CCl_4_ solution (40% in olive oil, the first dosage 0.5 mL/100 g, the others 0.3 mL/100 g) twice a week for either 3, 6, or 9 weeks to induce different degrees of fibrosis. The control group received normal saline.

### *In vitro* US imaging

The function of cRGD-PLGA-PFOB NPs as US contrast agents was imaged *in vitro* with a commercial US imaging system (7.5 MHz, L12-5 clinical US probe, iU22, Philips, Amsterdam, Netherlands). Images were obtained at the same parameter settings to compare the grayscale level in a water-filled silicon tube with that when the tube was filled with NP suspensions. The contrast enhancement was evaluated as a function of different cRGD-PLGA-PFOB NPs concentrations (50, 25, and 12.5 mg/mL).

### *In vitro* cytotoxicity of PLGA-PFOB and cRGD-PLGA-PFOB NPs

NP cytotoxicity in the rat hepatocyte (BRL-3A) cell line was determined using a cell counting kit-8 (CCK-8) cell viability assay (Dojindo Molecular Technologies, Kumamoto, Japan) [47]. NP suspensions were sterilized under ultraviolet light for 30 min before experiments. The BRL-3A cells were first seeded into 96-well plates at a density of 5.5 × 10^3^ cells per well and incubated at 37°C in a 5% CO_2_ atmosphere for 24 h. Wells without cells acted as blank controls. Different concentrations of PLGA-PFOB NPs and cRGD-PLGA-PFOB NPs in a solution medium (20, 10, 5, 2.5, 1.25 mg/mL) were prepared to replace the previous medium. After 24 h, 10 μL CCK8 reagent was added to each well and incubated for 4 h at 37°C. The absorbance of each well was measured at 450 nm with a microplate reader (EL×800 Universal Microplate Reader, BIO-TEK Instrument Inc, Winooski, VT, USA). All experiments were performed in triplicate.

### Cell-targeting ability of cRGD-PLGA-PFOB NPs in vitro

HSC-T6 and BRL-3A cells were maintained in Dulbecco’s Modified Eagle’s Medium (DMEM) (Gibco, Grand Island, NY, USA) supplemented with 10% fetal bovine calf serum (Gibco) and 1% penicillin-streptomycin at 37°C with 5% CO_2_. The HSC-T6 cells and BRL-3A cells were seeded onto glass-bottom Petri dish at a density of 1 × 10^6^ cells/mL. When HSC-T6 cells reached the log phase of growth at 60-70% confluence, they were incubated with 2 mg/mL targeted cRGD-PLGA-PFOB NPs for 30 min. After incubation, the culture medium was removed. The HSC-T6 cells were washed with PBS (pH=7.4) three times (3 min each) to remove extracellular cRGD-PLGA-PFOB NPs or free dye. The cells were fixed with 4% paraformaldehyde for 15 min and stained with 4’6-diamindino-2-phenylindole (DAPI) for 15 min before imaging. To prepare controls, PLGA-PFOB NPs without cRGD peptide were applied to HSC-T6 cells or cRGD-PLGA-PFOB NPs were applied to BRL-3A cells before being processed as described previously. Finally, a CLSM was used to observe the targeting ability of cRGD-PLGA-PFOB NPs.

### Acute biosafety of cRGD-PLGA-PFOB NPs

Prior to CCl_4_ solution injection, 6 rats received a dosage of 0.5 mL/100 g at cRGD-PLGA-PFOB NPs concentration of 50 mg/L via the caudal vein. Serum was sampled from the rats through a tail vein to detect biochemical indicators of liver and kidney function before and 1, 3, 7, and 14 days after cRGD-PLGA-PFOB NP injection. These biochemical indicators included total protein (TP), albumin (ALB), alanine aminotransferase (ALT), aspartate aminotransferase (AST), direct bilirubin (DBIL), total bilirubin (TBIL), blood urea nitrogen (BUN), and creatinine (SCr).

### Liver fibrosis stage analysis

The liver specimens were fixed with 4% formalin for 24 h and then embedded in paraffin. The liver sections were stained with hematoxylin-eosin (H&E) and Masson trichrome. The pathological fibrosis stage of the liver was analyzed by observing collagen distribution under a light microscope (Olympus DP27). For quantitative analysis, 10 fields (200×) from each section were randomly chosen and recorded. The Masson trichrome-stained (fibrotic) areas were measured using image analysis software (Image-Pro Plus 6.0, Media Cybernetics Inc., Silver Spring, MD, USA).

### Western blotting

The levels of rat integrin α_v_, β_3_, α-SMA, and TGF β_1_ expression were determined by western blot analysis. There is no specific antibody against rat integrin α_v_β_3_ available, but the integrin β_3_ subunit has been shown to bind to α_v_ (α_v_β_3_) or a_IIb_ (a_IIb_β_3_), and the latter is a membrane receptor expressed only in cells of megakaryocytic lineage and some tumor cells [48]. The rat liver tissues were lysed with radioimmunoprecipitation assay buffer on ice. The homogenate was centrifuged at 12,000 rpm for 10 min at 4°C, and the protein concentration was determined using bicinchoninic acid protein kit. Lanes were loaded with 100 μg of total protein, an immunoblot for glyceraldehyde 3-phosphate dehydrogenase (GAPDH) protein was used as a control for equal loading. Membranes were incubated with primary antibody (rabbit anti-rat integrin α_v_, 1:200, Boster, China; rabbit anti-rat integrin β_3_, 1:1000, Abcam, Cambridge, UK; rabbit anti-rat alpha smooth muscle Actin, 1:1000, Abcam; or rabbit anti-rat TGF β_1_, 1:1000, Abcam) overnight at 4°C. After washing, membranes were incubated with horseradish peroxidase-conjugated secondary antibodies and detected by chemiluminescence assay. GAPDH was included on the same film as an internal reference, and protein content was expressed based on the absorbance ratio of the target protein and the internal reference. A larger ratio indicated higher content of the protein of interest Immunoreactivity was quantified by densitometric analysis with Labworks^™^ Analysis Software (Lehi, UT, USA).

### *In vivo* US imaging and image analysis

A total of 48 rats were randomly divided into a control group (week 0), CCl_4_-treated 3-week group, CCl_4_-treated 6-week group, and CCl_4_-treated 9-week group (n = 12 per group). Each group was divided into a targeted group and a non-targeted group respectively (n = 6 per group). Animals in targeted groups were injected with cRGD-PLGA-PFOB NPs, while non-targeted groups were injected with PLGA-PFOB NPs. The rats were anesthetized with an intraperitoneal injection of 3% pentobarbital sodium solution at a dose of 0.1 mL/100 g. The agents (50 mg/L) were injected into rats via the caudal vein at a dose of 0.5 mL/100 g body weight. US imaging of the liver before and after cRGD-PLGA-PFOB NPs/PLGA-PFOB NP injection was performed by with a commercial ultrasonic diagnostic instrument (Esaote Mylab90, Florence, Italy) in a routine B mode. All imaging parameters were unchanged during the entire imaging experiment (frequency = 10 MHz, mechanical index = 0.7, depth = 44 mm, total gain = 76%). All liver images before and after injection were captured and stored. An Ultrasonic Quantitative Analysis Diagnostic System (invented by the Institution of Ultrasound Imaging of Chongqing Medical University, Chongqing, China) was used to quantitatively measure the EI according to the average of the gray-scale levels within the region of interest (ROI) in the liver parenchyma.

### Statistical analysis

Results are presented as the mean ± SD. Data were compared, and differences analyzed with one-way analyses of variance (ANOVAs) or Student’s *t*-tests with GraphPad Prism 7.0 (GraphPad Software Inc., La Jolla, CA, USA). A *P*-value less than 0.05 was considered statistically significant.

## CONCLUSIONS

In summary, we successfully developed an approach that could improve low sensitivity by providing a rational material design and novel strategy for efficient active-target US imaging of liver fibrosis using PLGA-PFOB NPs modified with cRGD peptides. The cRGD-modified PLGA-PFOB NPs showed high binding affinity and targeted efficiency to activated HSCs and exhibited excellent contrast-enhanced imaging capability for US molecular imaging, as demonstrated by *in vitro* studies and preliminary experiments in a rat model of liver fibrosis. The expression levels of integrin α_v_β_3_, α-SMA, and TGF-β_1_ increased with liver fibrosis progression. US molecular imaging with specific targeting of integrin α_v_β_3_ using cRGD-PLGA-PFOB NPs may successfully distinguish different stages of liver fibrosis, offering a novel and non-invasive method to monitor liver fibrosis progression.

## Abbreviations

CCl_4_: carbon tetrachloride
CLSM: confocal laser microscopy image
cRGD: cyclic arginine-glycine-aspartic acid octapeptide
ECM: extracellular matrix proteins
EI: echo intensity
FCM: flow cytometer
HSCs: hepatic stellate cells
NPs: nanoparticles
PFCs: perfluorocarbons
PFOB: perfluorooctyl bromide
PLGA: poly (lactic-co-glycolic acid)
α-SMA: alpha-smooth muscle actin
TGF-β1: transforming growth factor-beta 1
US: ultrasound
